# Cholesterol treatment with statins: Who is left out and who makes it to goal?

**DOI:** 10.1186/1472-6963-10-68

**Published:** 2010-03-17

**Authors:** Peter Franks, Daniel Tancredi, Paul Winters, Kevin Fiscella

**Affiliations:** 1 Center for Healthcare Policy and Research and Department of Family & Community Medicine, University of California at Davis, 4860 Y Street, Suite 2300, Sacramento, CA, 95817, USA; 2 Center for Healthcare Policy and Research and Department of Pediatrics University of California at Davis, 2103 Stockton Blvd., Suite 2224, Sacramento, CA 95817, USA; 3 Department of Family Medicine, University of Rochester; 1381 South Ave, Rochester, NY 14620, USA; 4 Departments of Family Medicine, Community & Preventive Medicine, Oncology, University of Rochester; 1381 South Ave, Rochester, NY 14620, USA

## Abstract

**Background:**

Whether patient socio-demographic characteristics (age, sex, race/ethnicity, income, and education) are independently associated with failure to receive indicated statin therapy and/or to achieve low density lipoprotein cholesterol (LDL-C) therapy goals are not known. We examined socio-demographic factors associated with a) eligibility for statin therapy among those not on statins, and b) achievement of statin therapy goals.

**Methods:**

Adults (21-79 years) participating in the United States (US) National Health and Nutrition Examination Surveys, 1999-2006 were studied. Statin eligibility and achievement of target LDL-C was assessed using the US Third Adult Treatment Panel (ATP III) on Treatment of High Cholesterol guidelines.

**Results:**

Among 6,043 participants not taking statins, 10.4% were eligible. Adjusted predictors of statin eligibility among statin non-users were being older, male, poorer, and less educated. Hispanics were less likely to be eligible but not using statins, an effect that became non-significant with adjustment for language usually spoken at home. Among 537 persons taking statins, 81% were at LDL-C goal. Adjusted predictors of goal failure among statin users were being male and poorer. These risks were not attenuated by adjustment for healthcare access or utilization.

**Conclusion:**

Among person's not taking statins, the socio-economically disadvantaged are more likely to be eligible and among those on statins, the socio-economically disadvantaged are less likely to achieve statin treatment goals. Further study is needed to identify specific amenable patient and/or physician factors that contribute to these disparities.

## Background

Disparities in the use of procedures by race, ethnicity, sex, and socioeconomic status (SES) have been widely documented including invasive cardiovascular interventions [1,2], joint replacement surgery [2,3], and other new or expensive technology [4,5]. These disparities may reflect either potential access (e.g. insurance or regular source of care) or realized access (visit number), [6] expenses (often not fully covered by insurance)[7] in addition to slower diffusion of innovation to minority patients or possibly to physicians and hospitals serving them [8-11].

In contrast, there is considerably less evidence for disparity in use of drugs for common, chronic conditions [12]. For example, there are few racial and ethnic disparities in inpatient medical management of congestive heart failure [13] and coronary artery disease [14] or in outpatient management of hypertension [15].

Statins may be an exception. They may be used less frequently among African Americans and poor patients [16-21] and these patients may be less likely to achieve target low density lipoprotein cholesterol (LDL-C) goal [22,23]. These findings have been variously limited by absence of cholesterol measurement, suboptimal SES measurement, and/or failure to fully apply the Third Adult Treatment Panel (ATP III) recommendations for treatment of elevated LDL-C.

Using recent US national data that included details on LDC-C levels, statin use, socio-demographic characteristics including race/ethnicity and SES, and healthcare access/utilization, three hypotheses were examined: 1) among persons not receiving statins, race/ethnicity, and lower SES would be associated with statin eligibility; 2) among persons currently receiving statins, race/ethnicity, and lower SES would be associated with lower likelihood of achieving LDL-C goals, and; 3) access/utilization related factors would attenuate these disparities.

## Methods

### Study Sample

Publicly available data were used from the continuous National Health and Nutrition Examination Surveys (NHANES) conducted between 1999 and 2006. NHANES is an ongoing survey designed to provide nationally representative estimates for the non-institutionalized population of the United States based on a complex multistage probability sample [24]. Survey data included household interviews, examinations, and testing. Following the interview, participants were invited to mobile examination centers. The protocol for NHANES was approved by the National Center for Health Statistics of the Centers for Disease Control and Prevention Institutional Review Board. Informed consent was obtained from each participant. During the study period, the response rate to the examination varied between 76-80% [25].

### Measures

Key independent variables were demographics (self reported age, sex, and race/ethnicity) and SES (household income [categorized as <100, 100-<150, 150-<200, 200-<300, 300-<400, and ≥ 400 percent of the federal poverty level] and highest educational attainment [<12, 12, and ≥ 12 years schooling]).

Statin use was based on a series of questions about prescription drugs reported taken during the previous month. For each drug reported, verification was obtained by asking the participant to show the medication container. Duration of use was also collected.

Statin eligibility and goal attainment were assessed using additional history, examination, and laboratory data. History factors included: coronary heart disease (CHD), myocardial infarction, stroke, angina, peripheral vascular disease, diabetes, cigarette smoking, use of antihypertensive medication, and family history of CHD.

Access/utilization factors included: language usually spoken at home (English vs. other); health insurance status (any vs. none), availability of usual source of care (any vs. none), and physician visits (0, 1, 2, 3, 4, or ≥ 5 per year).

Blood pressure was recorded as the average of up to two mercury manometer measurements (the first measurement was not included) obtained from the participant while sitting after a 5-minute rest [26]. Participants were classified as having hypertension if they had a systolic blood pressure of 140 mm Hg or more and/or diastolic blood pressure of 90 mm Hg or more and/or they reported currently using antihypertensive medication.

Blood samples were centrifuged and stored at -20°C and transferred to the Lipoprotein Analytical Laboratory at Johns Hopkins University in Baltimore, MD, for lipid analyses. Total cholesterol, high-density lipoprotein cholesterol (HDL-C), and triglycerides were measured using the Hitachi 704 Analyzer. LDL-C was calculated using the Friedwald equation if triglycerides were <400 mg/dl.

### Assessment for Statin eligibility and Target LDL-C

ATP III recommendations from 2004 for LDL-C management were used to assess statin eligibility and goal attainment [27,28]. Patients with no CHD or no CHD risk equivalents (diabetes, stroke or peripheral vascular disease) were scored on a count of major CHD risk factors (cigarette smoking, hypertension, low HDL-C [<40 mg/dl], family history of premature CHD, and older age [45 ≥ years for men; 55 ≥ y for women]); HDL-C 60 mg/dl or greater was considered protective and reduced the score by one. Participants with two or more risk factors underwent FRS to derive absolute 10 year CHD risk.

For adults with 0-1 risk factors, the statin eligibility threshold was an LDL-C ≥ 190 mg/dl with a treatment goal of <160 mg/dl. For adults with 2+ risk factors, the treatment goal was 130 mg/dl, with statin eligibility thresholds of 130 mg/dl for those with a 10-year CHD risk of 10-20% and 160 mg/dl if the risk was <10%. For participants with CHD, CHD risk equivalents, or FRS >20%, the statin eligibility and treatment goal for LDL-C were 100 mg/dl.

### Statistical Analyses

Descriptive, univariate, and regression analyses were conducted using STATA (version 10.1, StataCorp, College Station, TX), adjusting for the complex survey design of NHANES to yield population parameter estimates and appropriate standard errors. Logistic regression analyses examined the relationship between the key socio-demographic factors and: a) statin eligibility among those not on statins (the dependent variable was whether the respondent was eligible for statin therapy according to ATP criteria); and, b) goal attainment among those on statins (the dependent variable was whether the LDL-C level was at goal). To address whether access/utilization-related factors attenuated disparities, the analyses were conducted without and with adjustment for access/utilization-related factors. All analyses adjusted for study year. Regression results are presented as average marginal effects (AMEs), not adjusted odds ratios, to facilitate interpretation. The AME is the adjusted difference in prevalence of outcome (in %) between given category of predictor and its reference group.

Supplementary and sensitivity analyses examined model fit and explored possible explanatory pathways, including examining two-way interactions among independent variables, and adjusting for duration of statin therapy (< 1 year vs. ≥ 1 year), statin potency (atorvastatin or rosuvastatin vs. less potent [doses were not available]), and subject reported: prior testing for cholesterol; knowledge of cholesterol level; and doctor recommendation for treatment. Additional analyses excluded those with a history of liver disease (a possible contra-indication for statins).

## Results

Altogether, the NHANES surveys included 18,042 adults age 21-79. Data for FRS scoring was available on 15,260 (91% population weighted) of the sample. Persons with missing FRS information were more likely to be women (57% vs. 51%, p < .01), to have household incomes < 100% poverty level (17% vs. 12%, p < .01), and more likely (p < .01) to be African American (20% vs. 10%) or other race (7% vs. 5%). LDL-C levels were collected on a subset (N = 6913, population weighted = 45%) of those with FRS data; those without LDL-C were more likely to have household incomes < 100% poverty level (13% vs. 10%). Complete socio-demographic and LDL-C data were available on 6,580 persons. Of those with complete data, 537 (7.7%) reported current statin use. The characteristics of persons currently receiving compared to those not receiving statins are shown in Table 1; those not receiving statins were younger, poorer, disproportionately female, Black or Hispanic, lacked a usual source of care, had fewer visits, and were also more likely to have been surveyed in earlier years (p all < .01).

**Table 1:** Descriptive characteristics of sample (N = 6580), statin use, and statin eligibility among those not on statins (N = 6043).

	Total Sample	Proportion of total sample (N = 6580)	Proportion of total sample on Statins (N = 6580)	Proportion eligible of those not on Statins, (N = 6043)
**Total**	6580	1.00	0.08	0.10
**Age group**				
21-34	1,915	0.28	0.00	0.03
35-44	1,236	0.22	0.03	0.06
45-54	1,153	0.23	0.08	0.12
55-64	1,030	0.14	0.15	0.18
≥ 65	1,246	0.13	0.24	0.30
**Female**	3425	0.51	0.07	0.05
**Race/Ethnicity**				
White	3,267	0.72	0.08	0.11
Black	1,301	0.1	0.06	0.09
Hispanic	1,754	0.12	0.03	0.07
Other	258	0.05	0.11	0.12
**Federal Poverty Level**				
<100%	1,068	0.11	0.06	0.13
100-149%	926	0.10	0.07	0.12
150-199%	708	0.10	0.07	0.11
200-299%	1,048	0.16	0.09	0.11
300-499%	1,492	0.27	0.07	0.08
≥ 500%	1,338	0.27	0.08	0.10
**Education**				
<12 years	1,893	0.18	0.07	0.15
12 years	1,529	0.26	0.10	0.13
>12 years	3,158	0.56	0.07	0.08
**No Insurance**	1352	0.18	0.02	0.07
**Usual Source of Care**	5,549	0.85	0.09	0.11
**Physician Visits**				
0	1,093	0.16	0.01	0.08
1	1,203	0.2	0.03	0.08
2	1,735	0.28	0.09	0.11
3	1,550	0.22	0.13	0.13
4	473	0.06	0.11	0.15
≥ 5	526	0.08	0.12	0.09
**English Spoken at Home**	5,434	0.91	0.08	0.08
**Survey Year**				
1999-2000	1,385	0.21	0.02	0.12
2001-2002	1,720	0.26	0.03	0.11
2003-2004	1,687	0.26	0.12	0.11
2005-2006	1,788	0.28	0.12	0.09

Notes: Proportions are population weighted. Source: NHANES 1999-2006

Among the 6,043 participants not taking statins, 10.4% were eligible but not taking statins. Table 1 (last column) shows the factors associated with being eligible; these groups included: being older, male, non-Hispanic, poorer, less educated, uninsured, and having more visits (p all < .01).

**Figure 1:**
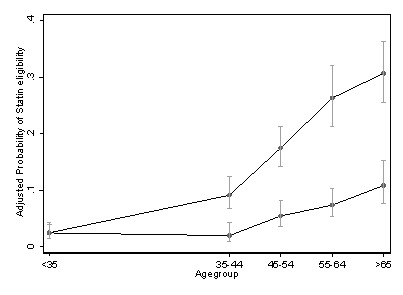
**Adjusted probability (and 95% confidence intervals) of statin eligibility among persons not taking statins, by age group and gender**.

Logistic regression analyses revealed the socio-demographic risk factors for being eligible (among those not taking statins) included being older, male, having lower income, and less education (Table 2). Hispanics were less likely to be eligible, an effect that became not significant after adjustment for the access/utilization variables (primarily language). None of the other socio-demographic effects observed were significantly attenuated by adjustment for the access/utilization variables. Among two-way interactions, the gender*age group effect was significant; the gender disparity in eligibility increased with age (Figure 1).

**Table 2:** Adjusted prevalence of statin eligibility among those not on statins (N = 6043), without (Model I) and with (Model II) adjustment for access/utilization.

	Model I				Model II			
**Social Risk Factor**	**AME**	**95% CI**		**P**	**AME**	**95% CI**		**P**

**Age group**								
21-34 (reference)								
35	7.66	3.11	12.21	<0.01	6.74	2.61	10.88	<0.01
45	18.96	11.72	26.20	<0.01	16.96	10.16	23.76	<0.01
55	29.84	21.76	37.93	<0.01	26.90	19.08	34.71	<0.01
≥ 65	42.48	34.32	50.65	<0.01	38.28	30.35	46.20	<0.01
**Female**	-11.54	-12.57	-10.50	<0.01	-11.69	-12.75	-10.63	<0.01
**Race/Ethnicity**								
White (reference)								
Black	-0.66	-1.81	0.49	0.26	-0.57	-1.54	0.40	0.25
Hispanic	-2.62	-3.74	-1.49	0.00	-1.65	-2.99	-0.30	0.02
Other	1.91	-1.48	5.30	0.27	2.28	-1.14	5.70	0.19
**Federal Poverty Level (%)**								
<100 (reference)								
100-149	-2.52	-4.86	-0.19	0.03	-2.22	-4.23	-0.21	0.03
149-199	-2.53	-5.76	0.70	0.12	-2.17	-5.01	0.66	0.13
200-299	-3.31	-5.54	-1.08	<0.01	-2.91	-4.90	-0.93	<0.01
300-499	-5.66	-8.82	-2.49	<0.01	-5.01	-7.80	-2.21	<0.01
≥ 500	-3.76	-5.46	-2.06	<0.01	-3.34	-4.79	-1.89	<0.01
**Education (years)**								
<12 (reference)								
12	0.10	-1.73	1.93	0.92	-0.05	-1.66	1.56	0.95
>12	-2.34	-3.90	-0.79	<0.01	-2.09	-3.48	-0.70	<0.01

Notes: AME = adjusted average marginal effect, adjusted % difference from reference group; CI = confidence interval. All analyses adjusted for survey year. Model II also adjusted for insurance, language, usual source of care, and visit number. Source: NHANES 1999-2006

**Table 3:** Descriptive characteristics of sample by statin use and goal attainment.

	Goal Attainment by Statin Use	
	Non-User N = 6043	User N = 537
**Total**	0.90	0.81
**Age group**		
21-34	0.97	1.00
35-44	0.94	0.83
45-54	0.88	0.88
55-64	0.82	0.76
≥ 65	0.70	0.80
**Female**	0.95	0.88
**Race/Ethnicity**		
White	0.89	0.82
Black	0.91	0.73
Hispanic	0.93	0.79
Other	0.88	0.82
		
<100%	0.87	0.54
100-149%	0.88	0.72
150-199%	0.89	0.79
200-299%	0.89	0.82
300-499%	0.91	0.88
≥ 500%	0.91	0.86
**Education**		
<12 years	0.85	0.78
12 years	0.87	0.81
>12 years	0.92	0.83
**No Insurance**	0.93	0.79
**Usual Source of Care**	0.89	0.82
		
0	0.92	0.69
1	0.92	0.84
2	0.89	0.80
3	0.87	0.80
4	0.85	0.82
≥ 5	0.91	0.87
**English Spoken at Home**	0.89	0.82
**Survey Year**		
1999-2000	0.88	0.62
2001-2002	0.89	0.77
2003-2004	0.90	0.83
2005-2006	0.91	0.83

Notes: Proportions are population weighted. Source: NHANES 1999-2006

Table 3 shows ATP III goal attainment among statin users and non-users. Among the 537 persons taking statins, 81.3% were at LDL goal. Goal attainment was less likely in men and poorer persons (p all < .01). The logistic regression analyses revealed that goal attainment was associated with being female and having higher income (Table 4); the effects of age, race/ethnicity, and education were not significant. There was no significant effect of the access/utilization variables. No interactions were statistically significant.

**Table 4:** Adjusted prevalence of being at LDL goal for those on statins (N = 537), without (Model I) and with (Model II) adjustment for access/utilization.

	Model I				Model II			
**Social Risk Factor**	**AME**	**95% CI**		**P**	**AME**	**95% CI**		**P**

**Age group**								
35-44	5.12	-17.93	28.17	0.66	4.26	-18.14	26.66	0.71
45-54	13.19	-4.48	30.85	0.14	11.59	-5.69	28.87	0.19
55-64	-5.49	-19.88	8.89	0.45	-4.63	-16.30	7.04	0.44
≥ 65 (reference)								
**Female**	14.07	8.95	19.20	<0.01	13.93	8.39	19.46	<0.01
**Race/Ethnicity**								
White (reference)								
Black	-6.38	-23.19	10.43	0.46	-4.91	-18.10	8.28	0.47
Hispanic	-1.83	-19.18	15.52	0.84	-2.33	-19.42	14.77	0.79
Other	3.70	-27.17	34.58	0.81	1.63	-25.83	29.10	0.91
**Federal Poverty Level (%)**								
<100 (reference)								
100-149	13.80	-4.26	31.87	0.13	13.84	-1.42	29.10	0.08
149-199	19.23	-1.77	40.23	0.07	18.02	1.65	34.39	0.03
200-299	26.63	11.96	41.30	<0.01	26.22	11.86	40.58	<0.01
300-499	28.50	15.50	41.50	<0.01	27.47	14.63	40.30	<0.01
≥ 500	31.54	20.80	42.29	<0.01	31.68	21.99	41.37	<0.01
**Education (years)**								
<12 (reference)								
12	-7.56	-24.18	9.07	0.37	-6.32	-20.02	7.39	0.37
>12	-4.95	-20.10	10.19	0.52	-3.89	-17.12	9.34	0.56

Notes: AME = adjusted average marginal effect, adjusted% difference from reference group; CI = confidence interval. Age group 21-35 omitted due to empty cells. All analyses adjusted for survey year. Model II also adjusted for insurance, preferred language, usual source of care, and visit number. Source: NHANES 1999-2006.

The supplementary analyses of statin eligibility among those not taking statins produced results similar to those presented. These models adjusted for patients reporting cholesterol testing (69%), patient knowledge of own cholesterol level (23%), and patient reporting as to whether the physician recommended treatment (8%); and, excluded those with a history of liver disease (3.7%).

Supplementary analyses of goal attainment examined the potential confounding effect of duration and potency of statin therapy. About 25% of respondents had been on statins for < 1 year, but there was no evidence that duration of therapy predicted goal attainment (<1 year, 78% vs. longer, 80%, p = 0.6). Statin potency was not significantly associated with goal attainment (p = 0.2), and did not affect the AMEs of the socio-demographic variables.

## Discussion

Analysis of this US nationally representative sample revealed significant disparities in the use of statins. Adults eligible, but not taking statins, were more likely to be older, male, of lower income, and less educated. The gender effect increased significantly with age. Among patients currently receiving statins, being male and having less income were associated with failure to attain LDL-C goal.

In contrast to previous US studies that used limited income adjustment [16-21], race/ethnicity was not significantly associated with statin eligibility or goal attainment; the study discrepancies suggest the key role of income.

Much of the US disparity literature has focused on race and ethnicity. These results underscore the significance of sex and SES on appropriate use of statins and on LDL-C goal attainment. Following a myocardial infarction men are *more *likely to receive appropriate interventions including angiography and revascularization [14]. However, men are more likely to be eligible, but not taking statins, and less likely to be at goal if treated. These findings, while contrasting with studies examining gender differences in the use of invasive procedures, are consistent with the notion that women may be more effective users of ambulatory care than men [29], and with findings that among those with high cholesterol women are both more likely to be aware of their condition [30] and more likely to be treated [21]. In addition, male sex and older age are included in FRS. This effectively lowers treatment thresholds among men and older persons, possibly contributing to sex and/or age disparities.

Among untreated persons, those with less income and less education are more likely to be eligible. These findings are consistent with previous studies showing that low SES is associated with less adequate care [12,31,32] including less statin initiation and persistence among those with lower income [33-35]. These effects may reflect barriers related to cost [36] and possibly patient beliefs [37].

There was little impact of access/utilization-related factors on disparities, other than for Hispanics. This may reflect a dominating effect of statin cost and lack of measures of usual source of *primary *care. At the time of this study, most statins were expensive and having insurance (including Medicare during this era) did not ensure prescription coverage. Even among those with insurance, higher copayments, and formulary restrictions deter statin use [38,39]. Furthermore, physicians consider formulary restrictions and patient out-of-pocket costs when making prescribing decisions [7,40]. However, it is worth noting that the findings on SES disparities are consistent with studies from countries with more equitable health systems (less socio-economically driven access barriers) including Australia [41] and Denmark[42]. Because these studies did not adjust for FRS, and lower SES is associated with a more adverse FRS profile [43] they likely under-estimate disparities in statin use. Taken together, these studies are consistent with research on the diffusion of new technologies, including statins, showing lower SES persons lag behind in the uptake of those new interventions [44].

The protective Hispanic effect, i.e. a lower likelihood of Hispanics being eligible, and its attenuation with adjustment for language is consistent with studies demonstrating Hispanics have better adjusted health status than Whites, a benefit that attenuates with greater acculturation [45-47].

Given the powerful effects of statins on cardiovascular and all-cause mortality, these results are concerning. Less optimal management of cholesterol among men and lower SES persons may contribute to higher CHD mortality [48]. The under-treatment of lower SES patients may exacerbate their risk for higher CHD mortality and contribute to widening disparities in CHD mortality [48]. In addition, because FRS does not fully account for the higher CHD risk in lower SES persons, fewer lower SES persons are identified for treatment than warranted [48]. Thus, lower SES persons may be at triple jeopardy: under-identified for treatment, undertreated once identified as eligible, and less likely to achieve treatment goals.

There are several potential explanations for the overall findings. Differences in statin eligibility among untreated patients could reflect physician and/or patient factors. However, an analysis adjusting for whether or not persons reported their physician recommended treatment produced similar results. The absence of an apparent effect of physician recommendation suggests that patient factors including acceptance of prescriptions for statins or differences in adherence barriers such as cost may contribute to higher rates of statin eligibility among the untreated. The overall low rate of physician recommendation among untreated patients (probably related in part to sequencing conditional questions), and potential bias in respondent recall of physician recommendations suggests caution in inferring an absence of physician contribution to these disparities. Further study is needed to determine the relative roles of patient factors (e.g. cost or beliefs) or physician factors (failure to adhere to guidelines) in contributing to these disparities. Doing so will facilitate the design of interventions to mitigate these disparities.

### Study Limitations

These finding are limited by reliance on cross-sectional data. The data provide no direct information on pretreatment cholesterol levels, drug doses, or number of times that physicians intensified therapy. Thus, the appropriateness of statin use among users cannot be precisely determined. For example, over-treatment of non-Hispanic Whites could have reduced the pool of untreated persons, masking racial differences. Variables selected to assess access/utilization may not optimally capture the underlying constructs. Thus, language usually spoken at home may not adequately capture access related to English proficiency; however, this variable performed similarly to one indicating interview language or use of interpreter. Last, we lacked data related to statin dose or cost. Some of those on statins achieving treatment goals may have been at goal without treatment. Limited sample size for some analyses, particularly assessing goal attainment for persons on statins, may have compromised power to detect significant effects.

## Conclusions

In conclusion, these US national data show that, among persons not taking statins, those with less income and men are more likely than their respective counterparts to be eligible, and, among those taking statins, these groups are also less likely to attain their LDL-C goal. Further study is needed to identify specific amenable patient and/or physician factors that contribute to these disparities.

## Competing interests

The authors declare that they have no competing interests.

## Authors' contributions

PF designed study, conducted analyses, interpretation of data and led writing. DT contributed to statistical analyses, interpretation of data and writing. PW contributed data preparation, interpretation of data and writing. KF obtained funding and contributed to writing. All authors read and approved the final manuscript.

## Pre-publication history

The pre-publication history for this paper can be accessed here:

http://www.biomedcentral.com/1472-6963/10/68/prepub
